# Photoprotection enhanced by red cell wall pigments in three East Antarctic mosses

**DOI:** 10.1186/s40659-018-0196-1

**Published:** 2018-11-21

**Authors:** Melinda J. Waterman, Jessica Bramley-Alves, Rebecca E. Miller, Paul A. Keller, Sharon A. Robinson

**Affiliations:** 1 Centre for Sustainable Ecosystem Solutions, School of Biological Sciences, University of Wollongong, Northfields Avenue, Wollongong, NSW 2522 Australia; 2 Department of Chemistry and Biology, University of Santiago, Alameda, 3363 Santiago, Chile; 3 School of Ecosystem and Forest Sciences, University of Melbourne, Richmond, VIC 3121 Australia; 4 School of Chemistry, University of Wollongong, Wollongong, NSW 2522 Australia

**Keywords:** Antarctic moss, *Ceratodon purpureus*, *Bryum pseudotriquetrum*, *Schistidium antarctici*, Bryophyte, UV-B-absorbing compounds, Cell wall, Anthocyanins, FT-IR

## Abstract

**Background:**

Antarctic bryophytes (mosses and liverworts) are resilient to physiologically extreme environmental conditions including elevated levels of ultraviolet (UV) radiation due to depletion of stratospheric ozone. Many Antarctic bryophytes synthesise UV-B-absorbing compounds (UVAC) that are localised in their cells and cell walls, a location that is rarely investigated for UVAC in plants. This study compares the concentrations and localisation of intracellular and cell wall UVAC in Antarctic *Ceratodon purpureus*, *Bryum pseudotriquetrum* and *Schistidium antarctici* from the Windmill Islands, East Antarctica.

**Results:**

Multiple stresses, including desiccation and naturally high UV and visible light, seemed to enhance the incorporation of total UVAC including red pigments in the cell walls of all three Antarctic species analysed. The red growth form of *C. purpureus* had significantly higher levels of cell wall bound and lower intracellular UVAC concentrations than its nearby green form. Microscopic and spectroscopic analyses showed that the red colouration in this species was associated with the cell wall and that these red cell walls contained less pectin and phenolic esters than the green form. All three moss species showed a natural increase in cell wall UVAC content during the growing season and a decline in these compounds in new tissue grown under less stressful conditions in the laboratory.

**Conclusions:**

UVAC and red pigments are tightly bound to the cell wall and likely have a long-term protective role in Antarctic bryophytes. Although the identity of these red pigments remains unknown, our study demonstrates the importance of investigating cell wall UVAC in plants and contributes to our current understanding of UV-protective strategies employed by particular Antarctic bryophytes. Studies such as these provide clues to how these plants survive in such extreme habitats and are helpful in predicting future survival of the species studied.

**Electronic supplementary material:**

The online version of this article (10.1186/s40659-018-0196-1) contains supplementary material, which is available to authorized users.

## Background

As the dominant flora of continental Antarctica, bryophytes (including mosses and liverworts) are extremely tolerant to harsh environmental conditions especially high ultraviolet radiation (UVR) levels, low temperatures, frequent freeze–thaw cycles and desiccation–rehydration events [1–6]. Recent climate change has significant implications for the survival of Antarctic bryophytes [7], with stratospheric ozone depletion since the 1970s producing a rapid increase in biologically damaging ultraviolet-B (280–315 nm; UV-B) light and stronger winds [8–10].

The three main mosses in the Windmill Islands, East Antarctica include two cosmopolitan species *Ceratodon purpureus* (Hedw.) Brid. and *Bryum pseudotriquetrum* (Hedw.) Gaertn, and the dominant Antarctic endemic *Schistidium antarctici* (Card.) L. Savic. & Smirn [7, 11]. These species produce and store UV-B-absorbing compounds (UVAC) within their cells and cell walls [12, 13], compounds that have high photoprotection value for Antarctic bryophytes surviving in physiologically extreme environments [14–17].

Water availability is the principal factor influencing the distribution of these species [18]; hence, moss beds in East Antarctica are limited to moist areas, typically around melt lakes that are fed by snow melt during the warmer temperatures in summer. Surrounded by water that freezes and thaws frequently throughout the summer (December to February) [4, 19], these moss beds experience small-scale frost heaving, which causes the formation of moss domes [20, 21]. Undulating moss turfs are common in coastal Antarctica, especially in the Windmill Islands region where moss landscapes of small peaks and valleys are found (Additional file 1: Figure S1). This microtopography causes microclimatic differences in temperatures and water availability as well as exposure to wind and radiation; all of which drive species health and distributions in the bryophyte community. For example, *C. purpureus* is characteristically found on drier crests and *S. antarctici* in the shallower, wetter troughs with *B. pseudotriquetrum* covering both microtopographical sites [11, 18, 22]. Green moss gametophytes growing in the depressed valleys tend to stay moist for longer whereas mosses situated on peaks exhibit drying and turn red or light brown in colour [17, 23]. This is especially apparent in *C.* *purpureus* where sections of turf exposed to the most light are ginger-red in colour and shaded moss turfs are bright green [17]. Transitions from green to red colouration in moss beds in the Windmill Islands region have been attributed to colder summers and windier conditions due to recent climate change [7]. Interestingly, the differences in specific compounds between red and green varieties have not been investigated for these Antarctic bryophytes.

Variation in moss colouration could be due to differences in carotenoid pigments, chlorophyll content, chloroplast movements, anthocyanin concentrations, UVAC or physical properties in cell layers [21, 24]. These variables are often investigated in vascular plants [e.g. 25, 26], mainly focusing on intracellular compartments, but are less commonly studied in bryophytes, despite colour being an important descriptive characteristic of the latter [24]. Bryophytes often exhibit red or brown pigmentation [24]. This could be due to pigments called chromatophores that occur in particular intracellular bodies such as vacuoles, bound to or in the cell wall [27 as cited in 28].

While many examples of red or brown phenotypes of bryophytes are described, only a few studies have successfully extracted and characterised the pigments of interest [28–30]. There are studies, however, suggesting that red pigmentation confers higher resilience to UV radiation than green [17, 31, 32]. For instance, Antarctic *C. purpureus* is known to vary its leaf pigmentation from green to red depending on the extent of increasing anthocyanin and decreasing chlorophyll concentrations [17]; however, the specific anthocyanins or other pigments causing this shift have not been isolated or identified. The red colouration could thus be present in this species simply as a side-effect of one or multiple abiotic stresses; or could be constitutively produced to protect the moss tissue from the harsh Antarctic environment. In addition, it is not known if there is a relationship between the localisation of UVAC and colouration in the three dominant East Antarctic bryophyte species.

This investigation examined changes in the UVAC within *C. purpureus, B.* *pseudotriquetrum* and *S. antarctici* when their red phenotypes were collected from the field and then grown under reduced light (no UVR). In addition, this study aimed to determine whether exposed *C. purpureus* (red form) has higher UVAC levels than shaded (green) moss. We hypothesised that field grown and red moss would have higher UVAC than laboratory grown or green forms. We also attempted to localise, extract and identify the pigment responsible for the red colouration in field samples of Antarctic *C.* *purpureus*.

## Results

### Pigmentation in adjacent red/green samples of field grown *C.* *purpureus*

**Fig. 1:**
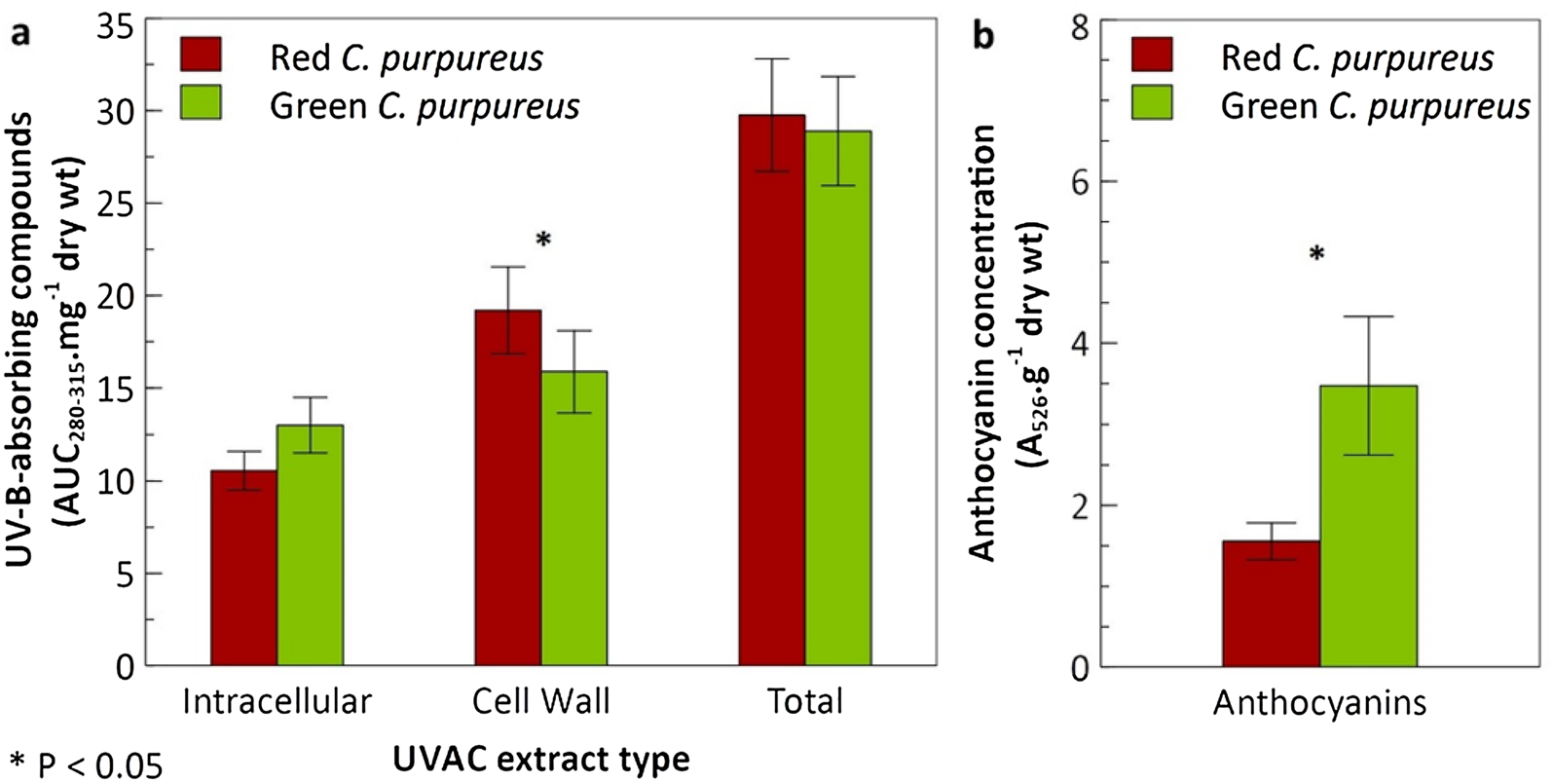
UV-B-absorbing compounds and anthocyanins in adjacent exposed (red) and shaded (green) moss samples. Comparison of mean total concentrations of **a** intracellular, cell wall and total UV**-**B**-**absorbing compounds are in terms of area under the curve between 280 and 315 nm (AUC_280–315_) mg^−1^ dry wt and **b** anthocyanin concentrations (n = 12 pairs). Bars are means (± SEM). Significant differences within extract types are marked with an asterisk. NB: Although the 1 SEM errors overlap for the cell wall bars, samples that are paired (and not independent) can show significant differences when the difference between them gives a small margin of error of its confidence interval. This consequently reflects a high correlation, which is taken into account in the statistics

Naturally occurring Antarctic *C.* *purpureus* red and green growth forms revealed different concentrations of both intracellular and cell wall UVAC but total UVAC were similar (Fig. 1a). The red type exhibited significantly higher levels of cell wall UVAC (matched pairs: t_11_ = 2.13, P < 0.05) whereas the green type had almost significantly higher intracellular UVAC concentrations (P = 0.068). Intracellular anthocyanins with absorbance at 526 nm were significantly more abundant in the green than the red paired samples (Fig. 1b; t_11_ = 2.0863, P < 0.05).

**Fig. 2:**
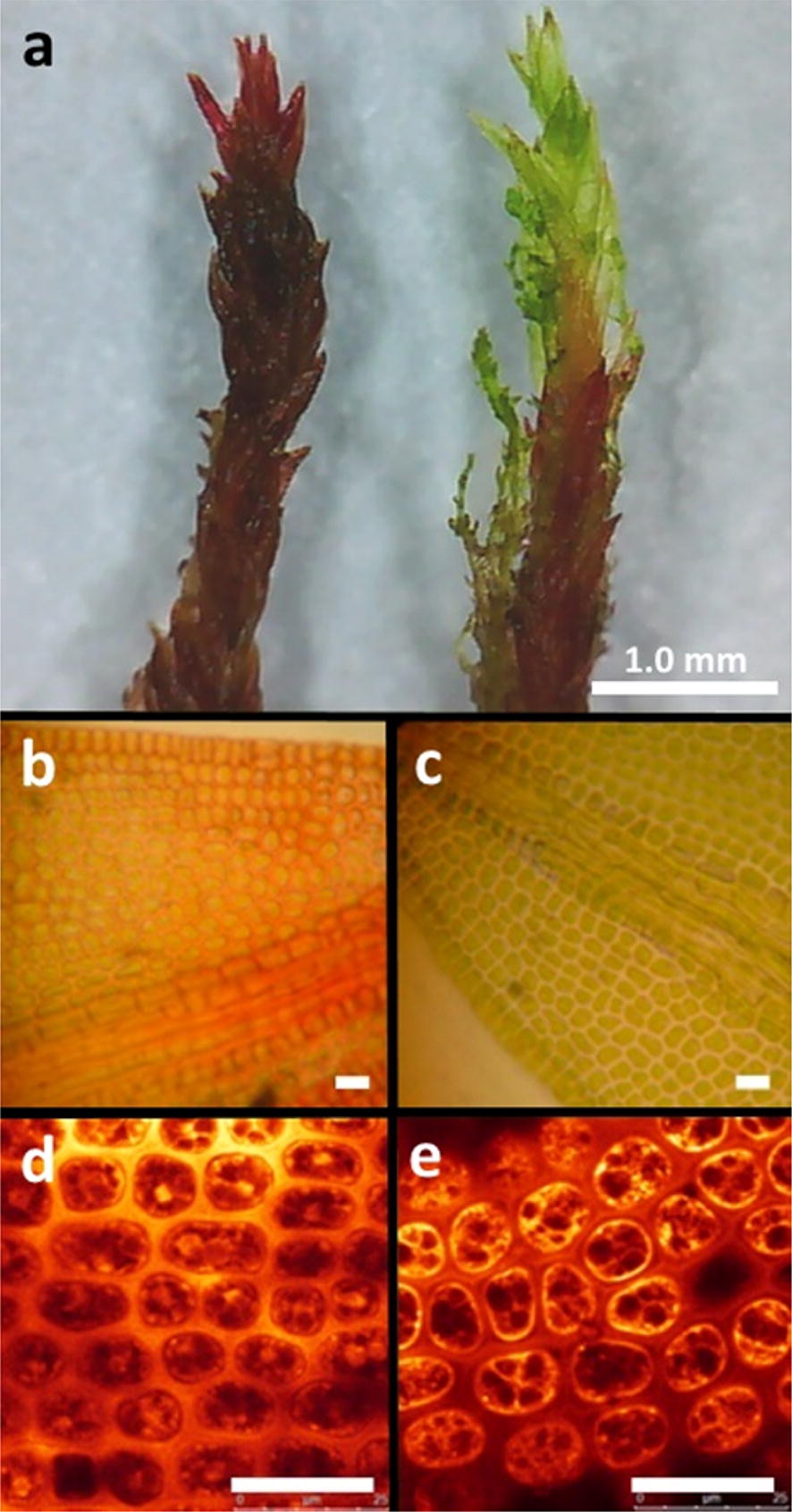
Colour and UV-B-absorbing compound localisation differences between exposed (red) and shaded (green) Antarctic *C. purpureus.* **a** Photographs of red and green gametophyte photosynthetic tips. Light microscopy images of **b** red and **c** green leaves. Confocal microscopy fluorescence images of **d** red and **e** green leaves stained with Naturstoff reagent A to visualise the location of UV-B-absorbing compounds. Yellow/orange fluorescence indicates the presence of phenolic compounds. Scale bars in **b**–**e** are 25 μm

Qualitative microscopic analysis revealed that the intense red colouration exhibited in this moss was associated with the cell wall (Fig. 2a, b). All leaves of *C. purpureus* examined, whether red or green, showed numerous healthy and green chloroplasts within the cells (Fig. 2b, c). The green colour from the chloroplasts was more pronounced in the green leaves, where the cell walls appeared to be colourless, but was masked by the red cell walls in the red growth form.

The localisation of phenolic compounds was further analysed using confocal microscopy. More intense fluorescence was detected in the cell walls of the red leaves than in intracellular compartments, which indicated a higher concentration of phenolics bound to the cell walls (Fig. 2d). Conversely, there was relatively more intracellular than cell wall fluorescence in the green leaves (Fig. 2e). Differences in specific compounds extracted from the cell walls of the red and green samples could not be established via HPLC analysis as most of the higher concentration peaks were poorly resolved and appeared to co-elute with the injection peak. Attempts to optimise separation by HPLC were unsuccessful.

### Fourier-Transform InfraRed (FT-IR) microspectroscopy analysis

FT-IR microspectroscopy was employed to detect differences in the cell wall architecture between red and green varieties of *C.* *purpureus*. Intense signals from cellulose polymer linkages (wavenumbers of < 1200 cm^−1^ and 1300–1400 cm^−1^) and phenolic vibrations (1640–1800 cm^−1^) were identified in the averaged spectra for both sets of leaves (Table 1 and Additional file 1: Figure S3). Although the spectral signals obtained from the red cell walls were much weaker than the spectra for the green samples, there were some significant differences. Signals at wavenumbers of 1250 and 1723 cm^−1^ were significantly higher in the green than the red leaves. These were assigned to ester linkages and C–O vibrations of pectin, respectively, and these signals were evidently missing in the red cell walls (Table 1). The majority of the significant differences for the peaks at < 1145 cm^−1^ were assigned to differences in cellulose content where the resultant negative t-values indicated weaker cellulose signals from the cell walls in the green leaves. Similarly, the small relative absorbance peaks at 1209 and 1555 cm^−1^ were significantly lower for the green samples, but the nature of these is unknown. It is unclear whether the significantly higher cellulose and unknown absorbance in the red walls is an artefact of noise due to its weaker signals or if these signals represent more mature walls containing more cellulose and phenolic compounds rather than the pectin observed in the green leaves.

**Table 1:** Relative absorbance FT-IR spectra for cell walls in red and green leaves of *C.* *purpureus*

‘Red’ cell wall peaks (cm^−1^)	‘Green’ cell wall peaks (cm^−1^)	Type of bond	Assignments to cell wall components (typical wavenumber signal in cm^−1^)
885	880		–
900	910	β-anomeric C–O	Cellulose (900)
1033	1035	C–C stretch	Cellulose (1040)
1064	1060	C–O stretch	Cellulose (1060)
1155	1160	C–O stretch	Cellulose (1160)
1209	–	C–O stretch	–
–	1250	C–O stretch	Pectin (1243)
1323	1304	C–H_2_ stretch	Cellulose (1320)
1375	1375	C–H_2_ stretch	Cellulose (1367)
1420	1430	Benzyl C=C stretches	Phenol
1610	1630	C=C stretch	Non-esterified uronic acid (1600–1630)
~ 1640 (weak)	1645	C=O stretch	Phenolic ring (1630)
–	1723	C=O stretch	Phenolic ester (1720)

Known cell wall components have been assigned to peaks in FT-IR spectra (Additional file 1: Figure S3) according to data reported in Mouille et al. [33] and Alonso-Simón et al. [34]. Peak signals are in wavenumbers (cm^−1^)

### Seasonal changes in UVAC of red growth forms

**Fig. 3:**
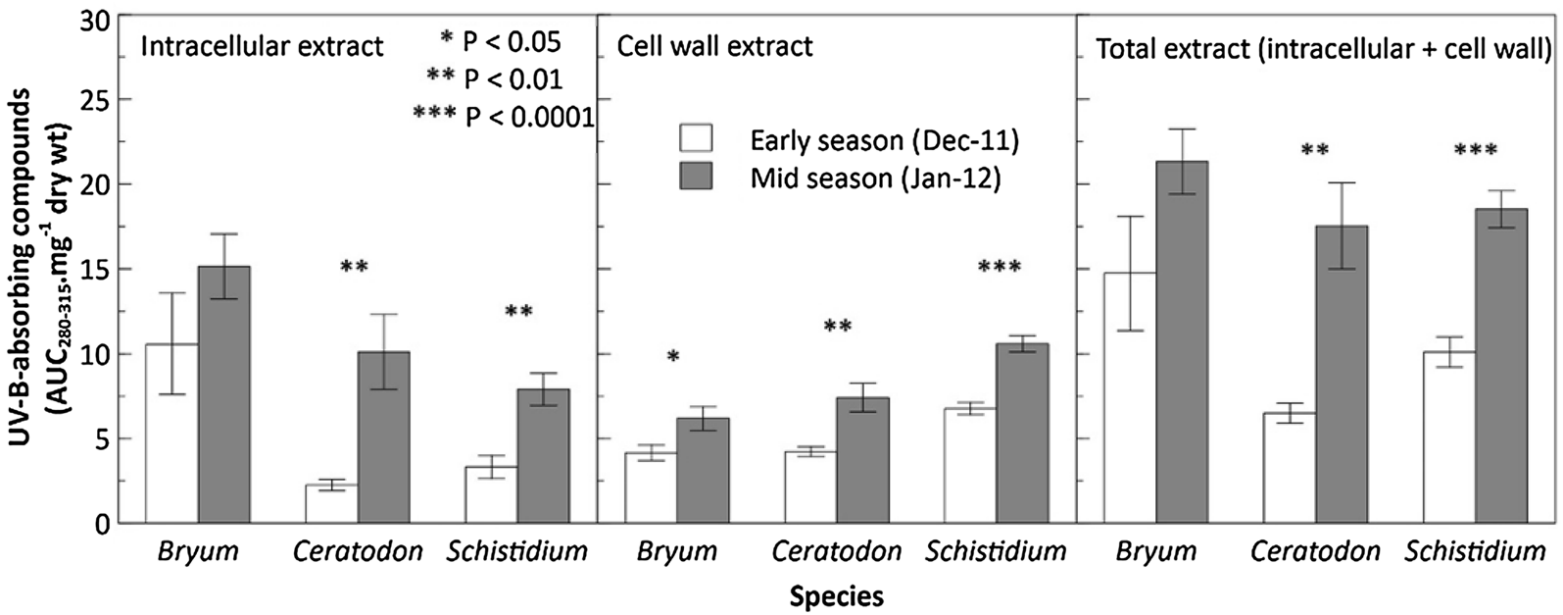
Intracellular, cell wall and total UV-B-absorbing compound concentrations for Antarctic *Bryum* *pseudotriquetrum*, *Ceratodon purpureus* and *Schistidium antarctici* collected at the beginning (December 2011) and middle (January 2012) of the austral summer season. Bars represent means (± SE). Significant differences within species are marked by asterisks (see Table 2)

Both intracellular and cell wall UVAC levels within red samples of all three species increased from early to midseason (Fig. 3 and Table 2). This was highly significant for intracellular, cell wall and total extracts obtained from *C.* *purpureus* and *S.* *antarctici* (P < 0.01) where concentrations had doubled within a month in their natural environment; but was only significant for the cell wall extract of *B.* *pseudotriquetrum* (P < 0.05; Table 2) when analysed individually via Student’s t-tests (not significant in the two-way ANOVA of all species).

**Table 2:** Statistical analysis of intracellular, cell wall and total UVAC concentrations for three red Antarctic mosses

	*B.* *pseudotriquetrum*			*C.* *purpureus*			*S.* *antarctici*		
	t stat	DF	P-value	t stat	DF	P-value	t stat	DF	P-value
Intracellular UVAC	1.34	12	0.205	3.52	10	< 0.01*	3.91	10	< 0.01*
Cell wall UVAC	2.23	12	< 0.05*	3.60	10	< 0.01*	6.32	9	< 0.0001*
Total UVAC	1.81	12	0.096	4.21	10	< 0.01*	6.02	9	< 0.0001*

Student’s t-test statistics and P-values (α = 0.05) showing differences between samples of Antarctic *B.* *pseudotriquetrum*, *C.* *purpureus* and *S.* *antarctici* collected from the field in early (Dec-2011) and mid growing season (Jan-2012; see also Fig. 3)

*DF* degrees of freedom

Significant P-values are marked with an asterisk

Species also showed significant differences independent of collection time. *Bryum pseudotriquetrum* had significantly higher concentrations of intracellular UVAC than both *C.* *purpureus* and *S.* *antarctici* (F_2,2_ = 5.91, P < 0.01) whilst the latter two species shared similar intracellular levels. Conversely, cell wall UVAC in *S.* *antarctici* were significantly higher than *C.* *purpureus* and *B.* *pseudotriquetrum* (F_2,2_ = 6.13, P < 0.01). The two cosmopolitan species also contained similar levels of UVAC within their cell walls. Consequently, the combined intracellular and cell wall UVAC concentrations (total) were significantly higher in *B.* *pseudotriquetrum* than *C.* *purpureus* with *S.* *antarctici* comparable to both (F_2,2_ = 4.04, P < 0.05).

### Greening under laboratory conditions

Red varieties of *B.* *pseudotriquetrum*, *C.* *purpureus* and *S.* *antarctici* collected midseason 2011/12 showed new, green growth during 2 weeks of optimal growth conditions in the laboratory. New green tissue grew in less than 7 days for *B.* *pseudotriquetrum*, approximately 7 days for *C.* *purpureus* and between 7 and 14 days for *S.* *antarctici*. This greening occurred in parallel with changes in UVAC amounts over the 2-week period.

**Fig. 4:**
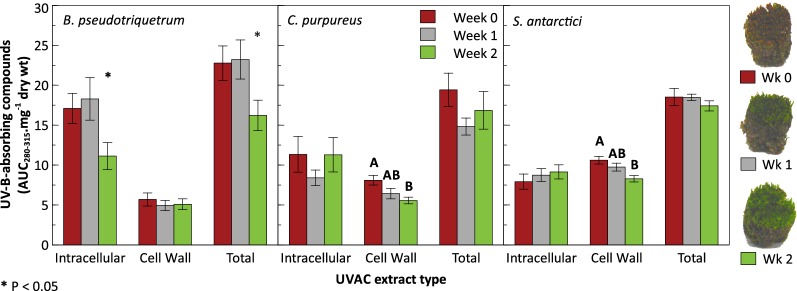
Mean (± SE) concentrations of UV-B absorbing compounds within intracellular and cell wall extracts of exposed (red) Antarctic *B. pseudotriquetrum*, *C. purpureus* and *S. antarctici* grown in reduced light, with hydration and warm temperatures for 2 weeks in the laboratory (n = 6). Bars within extract type that are not connected by the same letter are significantly different (Table 3). Asterisk indicates a significant difference at P < 0.05 where post hoc tests showed no significant difference

*Ceratodon purpureus* and *S.* *antarctici* exhibited significantly lower cell wall UVAC concentrations after 2 weeks but did not significantly alter intracellular UVAC (Fig. 4 and Table 3). Therefore, the apparent declining trend in total UVAC was insignificant. In contrast to *C. purpureus* and *S. antarctici*, *B.* *pseudotriquetrum* significantly decreased its intracellular UVAC concentrations, which contributed to a significant decline in total UVAC over the 2-week greening period. Cell wall UVAC levels (weeks 0–2) were maintained in *B.* *pseudotriquetrum* and seemed to be unaffected by new growth. Intracellular UVAC were significantly more abundant than cell wall UVAC for *B.* *pseudotriquetrum* (t_38_ = 6.82, P < 0.0001) and *C.* *purpureus* (t_34_ = 3.08, P < 0.01) but not for *S.* *antarctici* when weeks were pooled.

**Table 3:** Repeated measures ANOVA of UVAC concentrations for three red Antarctic mosses grown in the laboratory

	*B.* *pseudotriquetrum*		*C.* *purpureus*		*S.* *antarctici*	
	F-stat	P-value	F-stat	P-value	F-stat	P-value
Intracellular UVAC	F_2,10_ = 5.83	< 0.05	ns		ns	
Cell wall UVAC	ns		F_2,8_ = 5.93	< 0.05	F_2,10_ = 10.17	< 0.01
Total UVAC	F_2,10_ = 4.86	< 0.05	ns		ns	
Anthocyanins	ns		ns		n/a	

Statistical results (F-statistics and P-values where α = 0.05) are of intracellular, cell wall and total UVAC concentrations of Antarctic *B. pseudotriquetrum*, *C. purpureus* and *S. antarctici* that were grown for 2 weeks under low light and at 18 °C with adequate water (see Fig. 4). The repeated factor tested was for week (n = 6 for *S. antarctici* and *B. pseudotriquetrum*; n = 5 for *C. purpureus*)

*ns* not significant

**Fig. 5:**
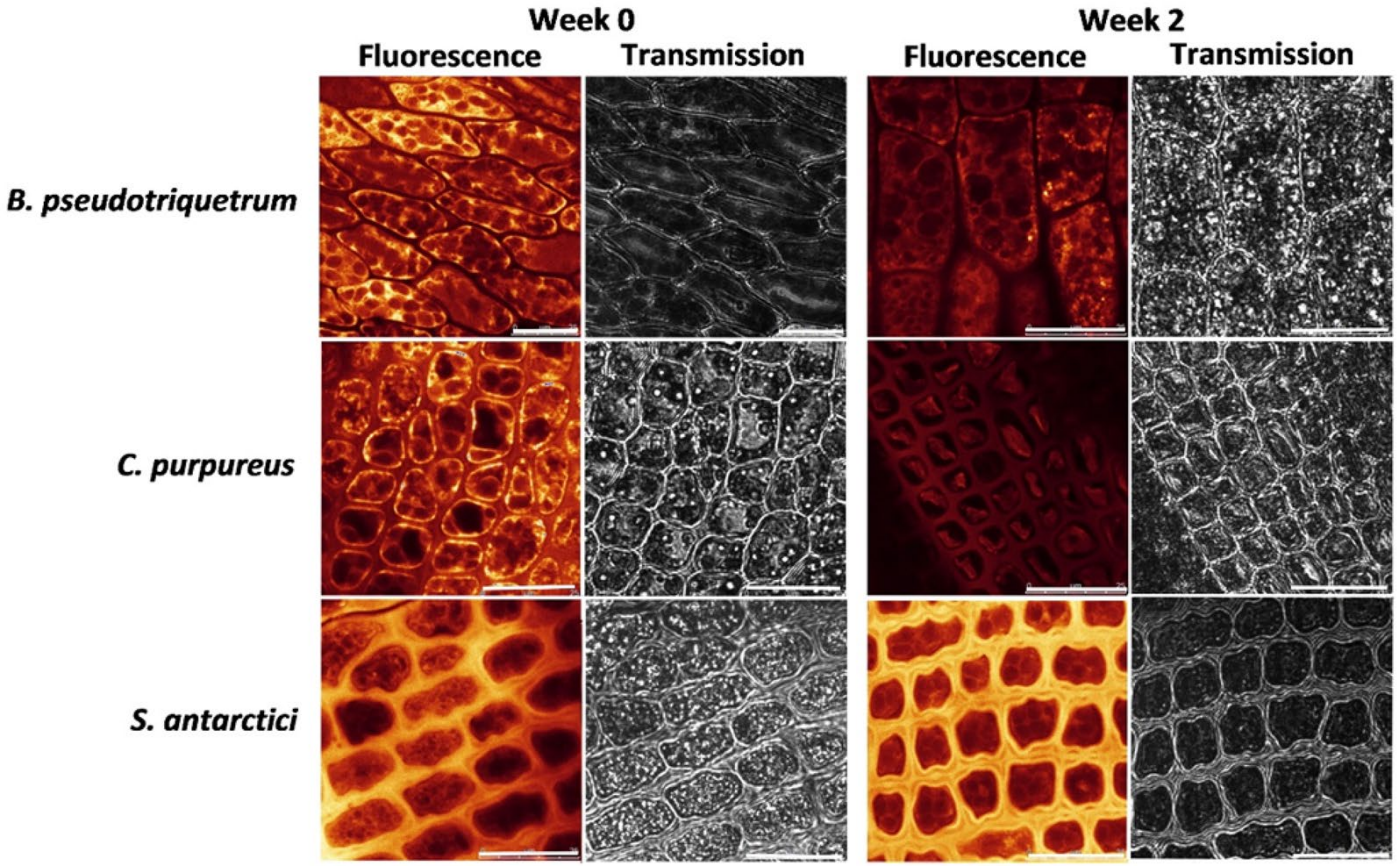
Confocal fluorescence and transmission images showing qualitative concentrations (fluorescence intensity) and location of phenolic compounds within cells and cell walls of red varieties of Antarctic *B.* *pseudotriquetrum*, *C.* *purpureus* and *S. antarctici* at week 0 and after 2 weeks of growth in the laboratory (green samples, conditions as in Fig. 4). Leaves were stained with Naturstoff reagent A and fluorescence images were captured in the 500**–**530 nm emission window under the same confocal settings. Yellow/orange fluorescence indicates the presence and concentration of phenolic compounds. Scale bars are 25 μm

Confocal microscopy and Naturstoff reagent A stain were used to localise phenolic compounds and the resultant images confirmed the above findings. *Bryum pseudotriquetrum* showed less fluorescence localised to the cytoplasm in week 2 samples than in week 0 (Fig. 5): however, there was a lack of fluorescence detected from the cell walls. Leaves of *S.* *antarctici* consistently showed relatively greater fluorescence from the stained cell walls than intracellular compartments, indicating higher concentrations of phenolic compounds localised to the cell walls. Levels of intracellular and cell wall fluorescence appeared to reduce in *C.* *purpureus*, which showed relatively low cell wall intensities at week 2 although the total signal was much weaker than at week 0.

## Discussion

This study has revealed that naturally red, exposed growth forms of Antarctic *C.* *purpureus* have higher levels of cell wall UVAC and lower intracellular UVAC as well as anthocyanin concentrations than its green, more shaded growth form. However, both colour morphs contained similar total UVAC concentrations. Also, anthocyanin trends described here confirmed those reported in Robinson et al. [35] which were the reverse of a previous study [17], although similar extractions were undertaken. Upon further analysis the intense red colouration of this species was shown to be associated with the cell walls rather than being localised in vacuoles or other intracellular compartments. FT-IR spectra indicated these red cell walls lacked phenolic ester and pectin signatures that were otherwise present in spectra obtained from colourless cell walls of *C.* *purpureus* green leaves, although both contained strong signals that represented cellulose and phenol or aromatic compounds. In addition, there were no peaks that could distinguish differences in the compounds between extracts from the red and green shoots via HPLC analysis. Therefore, the pigment responsible for the red colouration in the cell wall of *C.* *purpureus* is yet to be identified.

From early (December 2011) to midseason (January 2012) red varieties of *C.* *purpureus* exhibited a dynamic increase in all UVAC concentrations, which was found to be significant for intracellular and cell wall UVAC. Consequently, total UVAC more than doubled in this species over the duration of this experiment. Similar significant results were found for red samples of *S.* *antarctici* for all extracts. By contrast, the species *B.* *pseudotriquetrum* seemed to significantly increase only its cell wall UVAC. UVAC concentrations declined for all species when the midseason red-brown morphs were grown in a low light, warmer and hydrated environment but species varied in the cellular location of this change. A significant decrease was observed in the cell wall UVAC concentrations for both *C.* *purpureus* and *S.* *antarctici* over the 2 weeks of growth. In contrast, *B.* *pseudotriquetrum* reduced its intracellular UVAC. After 2 weeks, all moss species showed healthy new, green growth suggesting the three Antarctic species were thriving under these conditions.

### Cell wall UVAC are an important investment in exposed moss

Although significant differences in intracellular and cell wall UVAC were shown between the red (exposed) and green (shaded) Antarctic *C. purpureus*, these seemed to offset each other essentially resulting in similar combined UVAC levels (Fig. 1). This suggests *C. purpureus* growing in exposed sites produces the same total amount of UV-B-absorbing compounds as in shaded areas but these resources are integrated into a possibly more effective protective barrier in the cell wall rather than in the cytosol or intracellular compartments. The ability of *C.* *purpureus* to avoid UV-induced DNA damage in its desiccated state has been attributed to UVAC bound to its cell walls [1, 12], which is considered a better direct first defence against damaging UV rays than an intracellular location.

In Antarctic moss beds, desiccation events are more likely to occur for moss situated on ridges and in wind-exposed turfs where water is scarce and where exposure to high photosynthetically active radiation (PAR) and UVR is more likely than in shaded locations. Consequently, this microclimate subjects the moss to photosynthetically-stressful conditions [17], which appear to influence the localisation of the similar pool of UVAC. As the leakage of cytosolic solutes from cells can be quite substantial whilst moss is desiccating [36, 37], the cell wall is likely to be a better location in order to prevent loss of UVAC as long as the wall integrity is not compromised during desiccation. Antarctic mosses could localise these particularly important molecules within the cell walls as a preservation strategy where the compounds are less likely mobilised or leached during desiccation processes, thus preparing the tissue for other stresses like high UV light. This distribution of UVAC between cellular locations may also be affected by low temperatures and tissue age [38]. Hence, Antarctic *C.* *purpureus* moss might constitutively accumulate important UVAC in its cell walls ensuring protection against high radiation and desiccation.

### The photoprotective strategy of red cell wall pigments

Red pigments may also be produced and incorporated in the cell walls in order to physically protect against excess visible light. This physical barrier would effectively mediate faster recovery of photosynthesis when dried moss has been rewetted by reducing the formation of reactive oxygen species and protecting the chloroplasts from photobleaching [39]. For example, red gametophytes of a liverwort *Jamesoniella colorata* recovered faster than the green morphs upon rehydration showing a higher degree of tolerance to desiccation [40]. The red liverworts were also better protected from oxidative damage during the rehydration process. Red growth forms of *C.* *purpureus* may also show similar characteristics to this liverwort and could be better prepared to recover from desiccation than the green, shaded moss.

The red colouration in the walls of *C.* *purpureus* may be reducing light stress resulting in similarly healthy chloroplasts to the green form, as was detected via light and confocal microscopy (Fig. 2). The red pigments may act as photoprotective barriers by directly absorbing more PAR than the green leaves in a comparable way to *J. colorata* and another liverwort *Isotachis lyallii* [41]. The red morphs of these liverworts absorbed more green and blue wavelengths than their green counterparts but the green leaves absorbed and reflected more red and far-red light. In addition, the authors found that the red leaves of these liverworts had higher carotenoids than the green leaves, which was similarly the case for exposed moss on microtopographic ridges in Antarctica [21]. This suggests that Antarctic mosses respond strongly to red light, a characteristic of many bryophytes [42]. It is possible that red light is an important signal for these mosses to indicate environments where PAR may be in excess so that they can enhance the production of cell wall red pigments as well as UVAC to protect existing tissue.

### Cell wall UVAC decline under low radiation

A significant reduction of cell wall UVAC occurred when *C.* *purpureus* and *S.* *antarctici* from exposed sites were grown in low light (Fig. 4). This contrasts with *B.* *pseudotriquetrum*, which showed a significant decrease in intracellular UVAC. During the 2 weeks of growth, the original brown or red coloured gametophytes produced new green tissue showing that the laboratory conditions used were favourable for growth. New tissue development, in parallel with a reduction in cell wall UVAC, suggests that these wall compounds are present at significantly lower concentrations in young tissue and are probably laid down towards the end of cell maturation [43]. In addition, cell wall UVAC could be induced as new cells/tissues are exposed to changes in radiation, water and/or cold temperature stresses during their formation. This could be tested in the field in a similar way to a previous investigation in the Antarctic liverwort, *Cephaloziella* *varians*, where the authors studied changes in the dark pigmentation upon prolonged placement and subsequent removal of UV-BR filters [30]. It would be interesting to compare the studied mosses, especially *C.* *purpureus*, in this same way to see how their colouration responds to changes in UV light, desiccation and/or cold temperatures.

*Bryum pseudotriquetrum* showed a more dramatic response in the production of intracellular UVAC than the other two moss species over the 2-week laboratory experiment (Fig. 4). The extent of change is reflected in the time taken for new growth to emerge. For example, *B.* *pseudotriquetrum* produced bright green tissue earlier than the other two mosses, consistent with this species faster growth rate observed in the field [5, 44, 45]. Consequently, *B.* *pseudotriquetrum* would be expected to show more distinct changes in its intracellular UVAC mobilisation and/or production than *C.* *purpureus* and *S.* *antarctici*. Comparably, more subtle changes would be expected to occur for the other two species due to their slower growth rates [5, 44, 45]. If conditions are favourable, then it is likely that Antarctic *B.* *pseudotriquetrum* will be more responsive to environmental changes in the field [as seen in 14] and reflect these in its intracellular UVAC, whereas *C.* *purpureus* and *S.* *antarctici* probably show steady, efficient accumulation of UVAC in the cell walls during their slower active growth periods.

### Stress increases red colouration in Antarctic mosses

Considering that red moss was found in exposed locations, which are affected by multiple stressors, and that the red-brown colouration was absent in new green growth thriving under less stressful conditions, it is reasonable to suggest that the red pigmentation is stimulated under stress. It is unclear at this stage whether one or more stressors are responsible and the response may be species-specific. Whilst Antarctic *C.* *purpureus* has often been found to exhibit red tissue [17, 46], as has temperate *B.* *pseudotriquetrum* [47], previously there were fewer reports of red *S.* *antarctici* in the Windmill Islands. Although, there have been increasing accounts of red-tipped *S.* *antarctici* in recent years [7, 48]. This apparent change in the endemic moss may be an indication that it is responding to increasing stress occurring as a result of changes to its microclimate.

The seasonal increase in UVAC for all species may be due to environmental stresses intensifying across the 2011/12 season (Fig. 3). These include high PAR, UVR, cold and drought stresses [17, 49, 50], which are generally common in Antarctic environments [5]. However, the mosses would need to have been sufficiently metabolically active to synthesise and store secondary metabolites including UVAC. This would require at least a short boost of fresh snow melt or possibly a longer period of rehydration to provide the carbon necessary for production of new compounds [30].

### In search of the red compound in *C.* *purpureus*

Red or reddish-brown colour in *C.* *purpureus* was distinctly associated with pigments in the cell walls and our findings did not indicate chloroplast movement or chlorophyll *a*/*b* content changes. A cell wall pigment location is rarely found in higher plants [26] but has been increasingly reported in bryophyte studies [17, 28–30, 32, 41, 51, 52]. Previous investigations of *C.* *purpureus* have reported the colouration, but have not localised the red pigment or extracted the UVAC [17]. Several detailed attempts have been made to extract red pigments from bryophyte cell walls but without much success [41, 51].

FT-IR microspectroscopic techniques revealed that cell walls in red and green leaves were mainly composed of cellulose and pectin, which is expected for mosses [53]. In addition, the discovery of phenolic esters in the green *C.* *purpureus* species was not unusual as similar hydrolysed compounds were isolated from the cell wall, namely *p*-coumaric acid, *trans*-ferulic acid and *p*-hydroxybenzoic acids [13]. These were in their carboxylic acid form after extraction and isolation, but FT-IR analysis showed that they naturally exist as esters. These isolates are probably covalently linked to the cellulose strands during cell wall manufacture. Although phenolic esters were not detected in the red cell walls, a strong presence of phenolic ring signals was observed for both red and green leaves tested. These could be flavonoid or anthocyanidin derivatives [27 as cited in 28].

The identity of the red compound/s within *C.* *purpureus* is unresolved and it could be because they are very tightly bound to the cellulose architecture of the cell wall—so tightly bound that they could be very difficult to remove [28, 54]. Our findings suggest that the coloured compounds are strongly bound and incorporated within the cellulose as structural building blocks rather than loosely associated to the cell wall via hydrophobic interactions that would otherwise allow easy extraction using acidified methanol solvents [29]. Additionally, phenolics in plant cell walls could also form complexes with larger aromatic compounds, such as anthocyanins, reinforcing their binding to the cellulose [55, 56]. Similar to this study, Hooijmaijers and Gould [41] found it difficult to identify red cell wall pigments in the liverwort *J.* *colorata*. In contrast, an anthocyanidin called riccionidin A was identified as the dark purple/black pigment in the cell walls of the Antarctic liverwort, *C. varians* [30], but this pigment, which was removed using acidified methanol, could have been highly abundant in vacuoles as well and/or been weakly bound to the cell wall. Thus, anthocyanins or coloured phenolic compounds could be responsible for the colouration of *C.* *purpureus*; however, this is not yet confirmed and requires further investigation.

Future studies into the extraction of this tightly bound red pigment in *C.* *purpureus* are likely to require harsher extraction solvents as the current technique either did not extract a sufficient amount of the compounds of interest for identification; or they existed in polymeric or complex forms in the extract that were unable to be separated. The current method involved saponification (alkali hydrolysis) of cell wall residue at room temperature. As proposed in the FT-IR analysis, this hydrolysis probably facilitated the conversion of cell wall bound esters to carboxylic acids for their removal. Other approaches could include: digesting cell wall carbohydrates further using enzymes such as cellulase, targeting cellulose extraction using diglyme-HCl first and alkali hydrolysis second, or heating during the extraction process. For example, alkali hydrolysis at 200 °C was necessary to extract three phenolics from red cell walls of *Sphagnum nemoreum* moss [52]. The use of harsher solvents and reaction conditions however, risks severely altering the natural structure of the chemical/s responsible for the red/brown pigmentation within any plant species. Although investigations into the red pigments in *B.* *pseudotriquetrum* and *S.* *antarctici* were beyond the scope of the present study, identifying the red compounds for all these species remains an important avenue to pursue.

## Conclusions

It was shown that Antarctic *C. purpureus*, *B. pseudotriquetrum* and *S. antarctici* have reduced cell wall UV-B-absorbing compounds when grown in favourable conditions such as low light. Similarly, higher concentrations of cell wall UV-B-absorbing compounds were observed in red compared to green growth forms of Antarctic *C.* *purpureus* collected from the field. Red colouration in *C.* *purpureus* was clearly due to red cell walls and not chloroplast movement or chlorophyll content. These experiments suggest that the synthesis of UV-B-absorbing compounds in *C.* *purpureus*, *B.* *pseudotriquetrum* and *S.* *antarctici* is enhanced by exposure to high light, as well as other stressful conditions, and these compounds are only localised within cell walls during wall maturation, probably when new growth is exposed to high UV radiation or other stress triggers. This suggests that these cell wall compounds have a long-term protective role in these moss species. Previous studies that have just used methanol-based extractions may have completely underestimated the quantity and the variety of compounds responsible for UV radiation, drought or high light tolerance in many species, especially given that red cell wall pigmentation is quite common in bryophytes. This work demonstrates the importance of investigating cell wall pigments in plants and suggests that they could be much more widespread and important than currently realised.

## Methods

### Sample collection and experimental design

Samples of *Ceratodon purpureus* (Hedw.) Brid., *Bryum pseudotriquetrum* (Hedw.) Gaertn and *Schistidium antarctici* (Card.) L. Savic. & Smirn were collected at Casey Station in the Windmill Islands region, East Antarctica (66°16.9′S, 110°31.5′E). Of these three species, only *S.* *antarctici* is endemic to Antarctica. Sampling was undertaken during the 2009/2010 and 2011/2012 austral summers under the Antarctic Treaty (Environment Protection) Act 1980, Permit number ATEP2-12-13-4046 issued by the Commonwealth of Australia, Department of Environment to Robinson. Mosses were identified to species level by Robinson, Bramley-Alves or Miller on site at Casey Station.

Small cores (5–8 mm in diameter) of adjacent red (exposed) and green (shaded) Antarctic *C.* *purpureus* were sampled on 10th February 2010. Green moss was naturally shaded under small rocks or in troughs of moss turfs whereas red moss was in more wind- and sun-exposed microclimates (see Additional file 1: Figure S2). Additional moss plugs (approximately 10 mm in diameter) of exposed (red) turf of all three species were sampled from the field on 28th December 2011 and 23rd January 2012 (n = 18). Two-thirds of the January 2012 samples were placed into 24-well clear non-lidded trays (VWR International, Australia) and grown for 2 weeks in low light (10 μmol photons m^−2^ s^−1^ PAR), at 18 °C in the laboratory with adequate, but not saturating, water. Health of the moss plugs was assessed daily using a mini-PAM portable chlorophyll fluorometer (Walz, Germany) to measure the maximum quantum yield of photosystem II (Fv/Fm) after 20 min dark adaption. Moss plugs showed healthy Fv/Fm values between 0.7 and 0.8 before the gametophyte tips were harvested at 0, 1 and 2 weeks. Harvested samples were air-dried before storage at − 20 °C for transfer, extraction and analysis at the University of Wollongong (UOW), Australia.

### Extraction of intracellular and cell wall UV-B-absorbing compounds

The harvested and air dried moss gametophyte tips underwent freeze-drying at UOW (Christ Alpha 1-2 LDplus, Germany) set at − 54 °C to ensure complete dryness. Dried samples (10–20 mg dry wt) were transferred to microcentrifuge tubes (1.5 mL), each containing a 3 mm tungsten carbide bead, and ground using a TissueLyser (Qiagen, Australia) at 30 Hz for 2 min. Extraction solvent volumes and incubation times differed between the 2010 and 2011/12 samples as they were extracted at different stages of method refinement. For the red/green paired samples (2010), intracellular compounds were extracted using 1 mL of 1% HCl in methanol (CH_3_OH) for 1 h. Subsequent extractions using CH_3_OH (0.5 mL × 4; 1 h) resulted in a total intracellular extract volume of 3 mL. For the 2011/12 samples, intracellular compounds were extracted using 1% HCl in CH_3_OH (1.5 mL) for 3 h on ice (vortexed every 30 min) then centrifuged. Supernatants (intracellular extracts) were collected and stored at − 20 °C before analysis.

The remaining moss pellets were sequentially re-suspended to wash and extract the cell wall UVAC using a method adapted from Schnitzler et al. [57]. This was performed using CH_3_OH (2× 1.0 mL), NaCl solution (1 M, 1.5 mL) for 15 min, then CH_3_OH (1.0 mL), CH_3_OH–CHCl_3_ (1:1, 1.5 mL) twice for 1 h, before washing with CH_3_OH (1.0 mL). The pellets were then air dried and extracted in NaOH (1 mL). After this alkali hydrolysis, cell wall extracts for the 2010 samples (1 mL, in NaOH) were neutralised to pH 5.0 using 70 μL of conc. formic acid. In contrast, cell wall extracts obtained from the 2011/12 growing experiment were neutralised to pH 5.0 by adding 300 μL of 2.4 M formic acid to 0.7 mL of extract. Differences in volumes were accounted for in calculations. All cell wall extracts were either measured immediately or stored at 4 °C before analysis.

### UV–Vis spectrophotometry and HPLC analysis

Intracellular and cell wall moss extracts were analysed using UV–Vis spectrophotometry to monitor any change in UVAC concentrations via integration of the absorbance curves within the UV-B range (AUC_280–315 nm_) per mg of dry weight [58]. Cell wall extracts taken from *C.* *purpureus* samples at weeks 0, 1 and 2 of the growing experiment were further analysed via high pressure liquid chromatography (HPLC) separation to test for differences in specific UVAC as previously described by our group [13].

To test whether there were differences in concentrations of anthocyanins, a pH differential method was employed [21, 59, 60]. Absorbance of intracellular and cell wall extracts at pH 1.0 and 5.0 were measured at 526 nm. For the adjacent red/green (2010) samples, 0.2 M sodium acetate buffer (pH 4.5; 700 µL) was added to 1 mL of supernatant from the intracellular extraction, while 1.0 mL of buffer was necessary to neutralise the 2011/12 extracts (0.7 mL in CH_3_OH) from pH 1.0 to 5.0. Bulk anthocyanin concentrations were also tested within the neutralised cell wall extracts but samples needed to be acidified from pH 5.0 to 1.0 using conc. HCl. However, absorbances were higher at pH 5.0 than pH 1.0 resulting in overall negative concentrations. Therefore, the presence of anthocyanins in the cell wall extracts of these species could not be determined via this differential pH method.

### Confocal laser scanning microscopy

Leaves from moss gametophyte photosynthetic tips were mounted on glass slides in a droplet (20 μL) of distilled water. An excitation wavelength of 488 nm was used on a Leica DMI6000B inverted microscope situated inside a temperature and humidity controlled chamber coupled to a Leica TCS SP confocal system (Leica Microsystems, Germany). The background and chlorophyll autofluorescence for each sample was assessed in the 500–530 nm and 650–720 nm emission windows, respectively. A droplet (20 μL) of 0.5% (w/v) Naturstoff reagent A (2-aminoethyl diphenyl borate; Sigma-Aldrich, Australia), which was prepared from stock solution immediately before use, was then added to the tissue for the detection of phenolic compounds [57]. All images were processed online using LAS AF v.2.6.1 software or offline with LAS AF Lite (Leica Microsystems). Chlorophyll autofluorescence was found to be higher in the greener mosses that had been growing for 2 weeks in laboratory conditions.

### Fourier-Transform InfraRed (FT-IR) microspectroscopy

Several leaves (5–10 leaves) were removed from the tips of paired red and green gametophyte shoots and placed in 1.5 mL microcentrifuge tubes containing absolute ethanol (500 μL). Tubes were heated to 70–80 °C for 1 h to remove chlorophyll after which the ethanol was replaced with Milli Q water while being careful not to lose sample material. NaOH (1 mL of 1 M) was added to half of the washed leaves and left to extract overnight in an attempt to extract the red-coloured pigment from the cell walls; however, this was unsuccessful for the intact leaves tested. Therefore, the ethanol-extracted leaves were pipetted into cells of a 96-well plate. Leaves of interest remained whole and were transferred onto a circular BaF_2_ slide (2 mm thickness), rotated to face down and excess water was removed using a tissue. These leaves were flattened by covering this slide with another BaF_2_ slide (square; 1 mm thickness) and left to air dry for 3 h to remove the potential for large water signals that would otherwise obstruct underlying peaks.

Intact leaves were then analysed using a Hyperion 3000 Fourier Transform Infra-Red (FT-IR) microspectroscopy (Bruker Optics, Germany) at the High Resolution Plant Phenomics Centre, Commonwealth Scientific and Industrial Research Organisation (CSIRO), Canberra, Australia. Sections of leaves were selected and scanned 16 times within the wavenumber range of 800–1800 cm^−1^ and at a resolution of 8 cm^−1^ using OPUS 7.0 FT-IR software (Bruker Scientific Instruments, Germany). Homogeneity within a leaf was checked through multiple measurements. The acquired absorption spectra were converted to text files via Unscrambler X (CAMO Software) and normalised in R using a script courtesy of Grégory Mouille (National Institute for Agricultural Research, Versailles, France). Normalised spectra were then averaged, smoothed and derivatised to the first Savitsy-Golay derivative via PeakFit v4.12 (Systat Software Inc.; see Additional file 1: Figure S3).

### Statistical analysis

Statistical differences between samples during the growing experiment were assessed through a Repeated Measures ANOVA (RMANOVA; JMP Pro 9.0.2; SASS Institute Inc., USA; confirmed using IBM SPSS Statistics v19.0.0, SASS Institute Inc., USA). Anthocyanin data were transformed using $y = \sqrt x$ to satisfy the assumptions of the RMANOVA. The multivariate model was used when Box’s *M* test showed no significance and the Wilks’ Lambda F test is reported. Otherwise when Box’s *M* test showed significance, the univariate approach was used and when assumptions for equal variances were not met using Mauchly’s sphericity test (P < 0.05), the corrected univariate Huynh–Feldt F-statistic was used as opposed to the unadjusted univariate epsilon value. When significant differences were found, post hoc analyses were performed using Tukey’s HSD or Games-Howell tests when variances were equal or unequal (tested using Levene’s test of equal variances), respectively. Paired red/green samples were analysed using Matched Pairs t-tests (JMP Pro 9.0.2; SASS Institute Inc.) and seasonal differences between samples of red moss were performed using two-tailed Student’s t-tests (JMP Pro 9.0.2). For the latter, a two-way ANOVA was used to test for species differences. Differences between red and green FT-IR spectra (DF = 20) were determined using an R script which ran a series of multiple Student’s t-tests as in Mouille et al. [33]. As such any data above the t-value threshold are significantly higher in the green (control) leaves than in the red.

## Additional file

**Additional file 1.** Additional figures.

## Abbreviations

FT-IR: Fourier Transform Infrared Spectroscopy
HCl: hydrochloric acid
HPLC: high performance liquid chromatography
CH_3_OH: methanol
NaOH: sodium hydroxide
PAR: photosynthetically active radiation
UV: ultraviolet
UVAC: UV-B-absorbing compounds
UVR: ultraviolet radiation
w/v: weight/volume
