# Supplementary material for: Photoprotection enhanced by red cell wall pigments in three East Antarctic mosses
Source: Biol Res. 2018 Nov 21;51:49. doi: 10.1186/s40659-018-0196-1 (PMC6247747; doi:10.1186/s40659-018-0196-1)
Supplement: Supplementary file 1 — Additional file 1. Additional figures. [file 40659_2018_196_MOESM1_ESM.docx]

Research Article for Biological Research

# Photoprotection enhanced by red cell wall pigments in three East Antarctic mosses

Melinda J. Waterman, Jessica Bramley-Alves, Rebecca E. Miller, Paul A. Keller, Sharon A. Robinson.

# Additional Figures


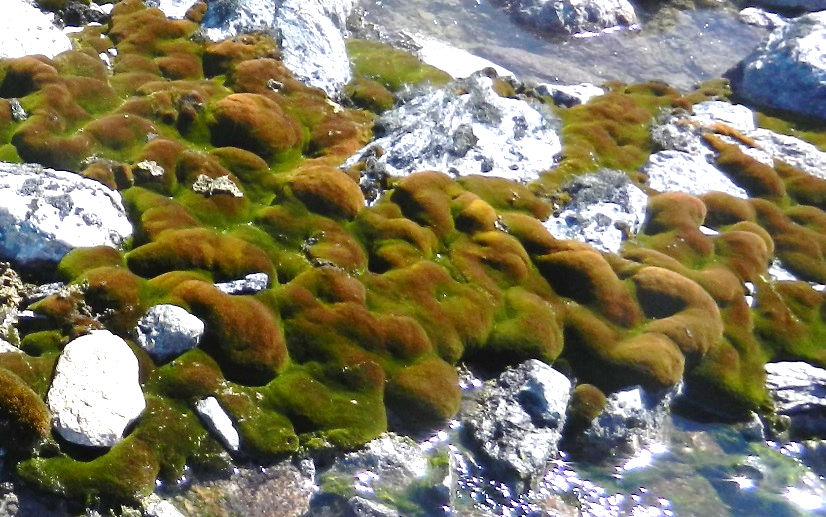


**Figure S1. A typical moss landscape in the Windmill Islands region, East Antarctica.** Here peaks in the moss bed are red-brown (likely to be *Ceratodon purpureus*) and green moss can be found in the valleys (typically *Schistidium antarctici*). Another species, *Bryum pseudotriquetrum* is found in both microtopographical . Photograph courtesy of Jenny Watling.


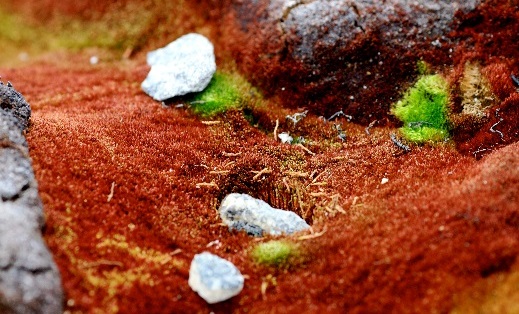

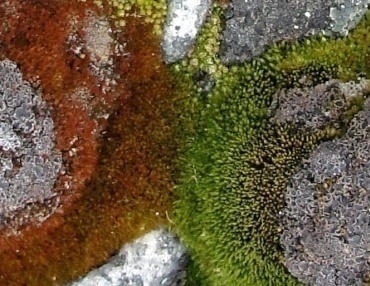


**a**

**b**

Figure S2. Examples of adjacent exposed (red) and shaded (green) moss in the field at Casey Station, East Antarctica. Green growth forms can be found under small rocks (a) or within valleys (b) that have provided varying levels of shading and/or protection from stressful conditions.


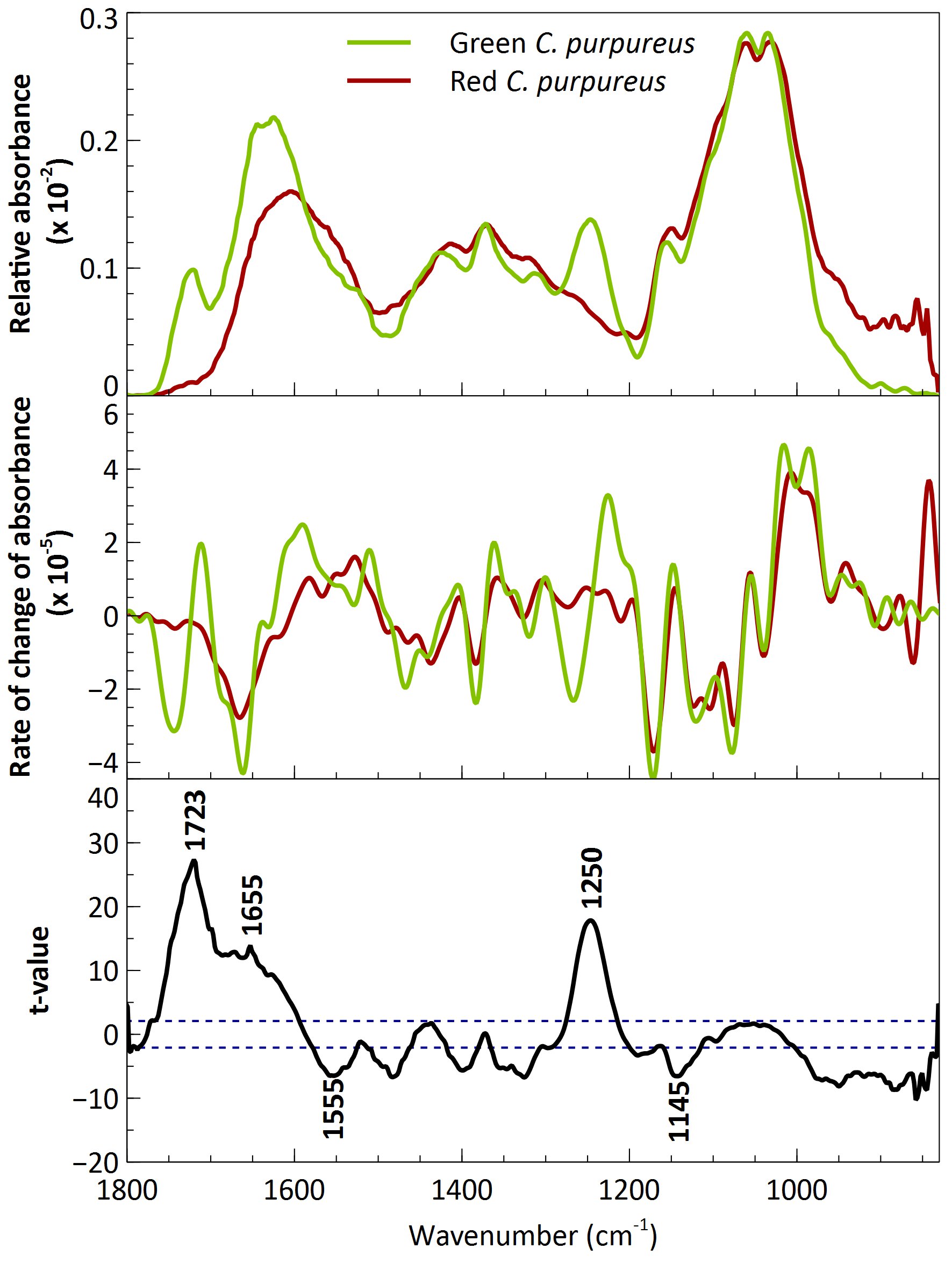


**c**

**b**

**a**

Figure S3. a Mean FT-IR spectra (22 in total) of cell wall sections from red (n = 10) and green (n = 12) leaves where the cell contents have been removed; b first derivatives of averaged spectra for both after Savitsky-Golay smoothing and normalisation; c t-values for the comparison between the relative absorbance spectra for red and green leaves plotted against the corresponding wavenumbers which indicate higher intensities in the green leaves (control) than the red leaves (treatment). Dashed horizontal lines represent the significance threshold at α = 0.05. Major significant peaks are assigned.
